# Airborne LTA Nanozeolites Characterization during the Manufacturing Process and External Sources Interaction with the Workplace Background

**DOI:** 10.3390/nano12091448

**Published:** 2022-04-24

**Authors:** Riccardo Ferrante, Fabio Boccuni, Francesca Tombolini, Claudio Natale, Daniela Lega, Alessandra Antonini, Sergio Iavicoli

**Affiliations:** 1 Department of Occupational and Environmental Medicine, Epidemiology and Hygiene, Italian Workers’ Compensation Authority, Via Fontana Candida 1, I-00078 Rome, Italy; f.boccuni@inail.it (F.B.); f.tombolini@inail.it (F.T.); 2 Research Centre on Nanotechnology Applied to Engineering (CNIS), Department of Astronautical, Electrical and Energy Engineering, Sapienza University of Rom, Piazzale Aldo Moro 5, I-00185 Rome, Italy; claudio.natale@uniroma1.it; 3 Department of Technologies, Italian Workers’ Compensation Authority, Via del Torraccio di Torrenova 7, I-00133 Rome, Italy; d.lega@inail.it (D.L.); al.antonini@inail.it (A.A.); 4 Directorate General for Communication and European and International Relations, Italian Ministry of Health, Lungotevere Ripa 1, I-00153 Rome, Italy; s.iavicoli@sanita.it

**Keywords:** nanozeolites, nanoparticles, nanomaterials, exposure monitoring, environmental pollutants

## Abstract

Engineered nanoscale amorphous silica nanomaterials are widespread and used in many industrial sectors. Currently, some types of silicon-based nanozeolites (NZs) have been synthesized, showing potential advantages compared to the analogous micro-forms; otherwise, few studies are yet available regarding their potential toxicity. In this respect, the aim of the present work is to investigate the potential exposure to airborne Linde Type A (LTA) NZs on which toxicological effects have been already assessed. Moreover, the contributions to the background related to the main emission sources coming from the outdoor environment (i.e., vehicular traffic and anthropogenic activities) were investigated as possible confounding factors. For this purpose, an LTA NZ production line in an industrial factory has been studied, according to the Organisation for Economic Cooperation and Development (OECD) guidelines on multi-metric approach to investigate airborne nanoparticles at the workplace. The main emission sources of nanoparticulate matter within the working environment have been identified by real-time measurements (particle number concentration, size distribution, average diameter, and lung-deposited surface area). Events due to LTA NZ spillage in the air during the cleaning phases have been chemically and morphologically characterized by ICP-MS and SEM analysis, respectively.

## 1. Introduction

In the last 20 years, nanotechnologies (NTs) acquired a key role in industrial sectors in which engineered nanomaterials (NMs) were broadly used, such as chemical, pharmaceutical, health, energy, electronics, textile, agri-food, and others [1,2,3,4].

NMs are defined by the International Organization for Standardization (ISO) [5] as materials with any external dimensions at the nanoscale (size range from approximately from 1 to 100 nanometers). The ISO also defines nano-objects and their agglomerates and aggregates (NOAA) as materials with one, two, or three external dimensions at the nanoscale; aggregates are strongly bonded or fused particles, whereas agglomerates are weakly bound particles.

The global market of NTs is expected to grow of about 17% per year in the period of 2018–2024 [6]. In the European context, NTs have been included among the key enabling technologies (KETs), as a fundamental tool to support industrial research and innovation of the European Commission’s Horizon 2020 [7] and Horizon Europe 2021–2024 programs. Simultaneously, the growing attention toward the responsible development of NTs was highlighted by the amount of scientific publications in the fields of environmental impact and safety and health at work [8]. In this framework, amorphous silica (SiO_2_) nanoparticles are widespread used in different sectors, from medicine and pharmaceutics to the construction industry [9] with an estimated commercial volume of 1,500,000 tonnage produced per year [10]. After all, silica is the most abundant mineral in the earth’s crust and this also reflects a great use of such materials in work environments. In particular, zeolites are nano-porous materials with pores ranging from 0.3 nm to 1 nm [11]; they are natural or synthetic crystalline materials and their atomic structures are based on three-dimensional frameworks of silica and alumina tetrahedra: AlO_4_ and SiO_4_ crystals, with silicon or aluminum ions surrounded by four oxygen ions in a tetrahedral configuration. Each oxygen is bonded to two adjacent silicon or aluminum ions, linking them together [12]. A three-letter code is assigned to such materials to more easily distinguish the different types [13]: as an example, Linde Type A zeolites are represented by the code LTA. Currently, nano-forms of some types of zeolites have been synthesized: these NZs show potential advantages compared to the analogous micro-forms, due to their increased external surface and reduced diffusion paths of the molecules within the individual crystals [14]. In nanomedicine, NZs are exploited for their high absorption and immobilization capacities due to their surface characteristics [15,16]. In indoor agriculture, due to their ability to retain water, they act as a slow-release water source, which also helps to minimize water waste [17].

In parallel with the production and use of NZs for different applications, the scientific community has focused its interest in the potential impact for workers involved in industrial and small-scale research and development (R&D) processes. Although it is well known that exposure to respirable amorphous and crystalline silica is widely studied [18,19,20,21,22,23] and exposure to silica particles may cause permanent lung scarring (pulmonary fibrosis) [24], few studies are available in the literature about the toxicological potential of NZs. In particular, Kihiara et al. (2011) [25] studied the in vitro toxicity for different types of NZs and concluded that cytotoxicity effects could be influenced by the shape and size of the aluminum crystal. In addition, aluminum-containing types of NZs—such as ZSM-5, LTL, and LTA—show dose-dependent toxicity. Thomassen et al. (2012) [12] investigated the cytotoxicity of NZs type A and Y: they concluded that these NZs exhibited very low toxicity, when compared to that of the amorphous silicon particles used as a positive control in their study. Męczyńska-Wielgosz et al. (2016) [26] studied NZs type A (BaA) functionalized with polymer materials (PEG) and demonstrated that their cellular uptake, retention inside the cell, and cytotoxicity in vitro depend on the molecular weight of PEG. Marziye Hejazy et al. (2018) [27] investigated their teratogenic and embryotoxic effects in chick embryos as a model for evaluating human embryonic damage, and they found teratogenic effects including deformity of legs, wings, liver, and heart.

In this framework, the aim of this work is to study the potential exposure to airborne Linde Type A (LTA) nanozeolites (NZs) during their production phases in order to identify the potential spills, as a contribution to investigate the potential impact on workers’ health. The exposure assessment, according to the guidelines proposed by the Organization for Economic Cooperation and Development (OECD) [28,29], is based on a multi-metric approach optimized for the case study. Furthermore, background contributions from external emission sources have been evaluated using chemical and physical characterization techniques.

This study was carried out within a larger research project in which the cyto-genotoxic and inflammatory effects of produced LTA NZs in human alveolar epithelial cells were also evaluated by Cavallo et al. (2020) [30] who reported mild but detectable early genotoxic and inflammatory effects. In this context, the present paper represents the first study on the characterization of occupational exposure to airborne LTA NZs on which toxicological effects have been already assessed.

## 2. Materials and Methods

The case study involved a factory in which LTA NZs were produced and stored, housed in an area of about 1300 m^2^ including buildings with 5 m height. LTA NZ production was about 10 kg per year and two workers were directly involved in powders handling phases.

The LTA NZ production process involves several phases. We focused our attention in five main production phases in which potential exposure of involved workers may occur: cleaning, synthesis, drying, surface modification, and activation. Table 1 reports the experimental details (i.e., processing, number of workers, and physical state of materials) related to the monitored work activities.

In terms of risk assessment and management, it is important to characterize airborne NMs potentially released in the workplace in order to identify the critical phases of the production cycle, as for example the handling of NMs in powder form or the cleaning procedures as previously described in literature [9,31]. The debate is still open as to how many and which parameters are needed to be considered for a comprehensive occupational exposure assessment and to better represent NMs toxicity [32,33,34,35,36,37,38]. Mass and number concentration are the most widely used parameters [39]; however, in many cases they are not enough to describe complex exposure scenarios [40].

Therefore, it is very important to choose the best parameters useful to quantify the exposure levels (i.e., number concentration, particle size, and surface area) and to characterize the physiochemical properties of NMs collected in the workplace air (i.e., shape and elemental composition).

In this case, the measurements were carried out with on-line and off-line instruments, allowing the monitoring of mass concentration (MC), particle number concentration (PNC), size distribution (SD), average diameter (D_avg_), and lung-deposited surface area (LDSA). In particular, LDSA represents a key parameter for occupational exposure, because it takes into account the deposition efficiency of airborne NMs in different compartments of the lung (TB: tracheobronchial; A: alveolar) [41]. LDSA depends on the real active particle surface and it is identified as the biologically most relevant dose metric for spherical nano-objects explaining about 80% of the observed variability in acute pulmonary toxicity [42].

An 8 h time-weighted average (8 h-*TWA*) has been calculated as time weighted deposited surface area concentration averaged for an 8 h period according to

(1) $$8h-TWA=\frac{\sum_{i}\left(C_{i}\times T_{i}\right)}{28,800}$$

where *C_i_* is the concentration (μm²/cm³) during the averaging interval *T_i_* (sec). The sum of all averaging intervals (*Σ_i_ T_i_*) or the total sampling period may be less, equal to or greater than 8 h; however, the time weighted concentrations are always averaged for an 8 h period (28,800 s).

In addition, particle bound polycyclic aromatic hydrocarbons (p-PAHs) surface-adsorbed on powders below 1 µm were monitored both during background and production phases, to take into account the potential formation of secondary organic aerosol (SOA) [43].

Microclimatic parameters—such as ambient temperature, atmospheric pressure, and relative humidity—were also observed using a BABUC A (LSI-Lastem Inc., Milano, Italy) with a time resolution of 1.5 min.

For on-line measurements instruments with high time resolution (1 Hz) were used; for off-line analysis, airborne materials were collected by inertial cascade impactors based on aerodynamic diameter, to evaluate size range, mass, chemical composition, and morphology of the airborne matter during LTA NZ production phases.

The experimental setup of instruments involved in the measurement campaigns is summarized in Table 2. The working principles of all the instruments used in the present study were discussed in detail elsewhere [44].

In the present study, a multi-metric approach based on OECD guidelines [28,29] has been chosen for the exposure assessment to airborne LTA NZs. The measurement strategy included:

1. Gathering information about workplace activity, physical and chemical properties of the NZs (summarized in Table 3), and exposure scenario by filling a questionnaire based on ISO, 2011 [5], addressed to the LTA NZs manufacturer.
2. Field investigations to conduct a basic exposure assessment using easy to use and portable equipment to measure airborne NMs by real-time measurements and sampling for further off-line analysis. Simulations on trial materials are also performed in a laboratory setting.
3. Expert exposure assessment using all appropriate equipment and available characterization techniques to provide a definitive conclusion regarding the presence of airborne nanomaterials in the occupational setting.

**Figure 1:**
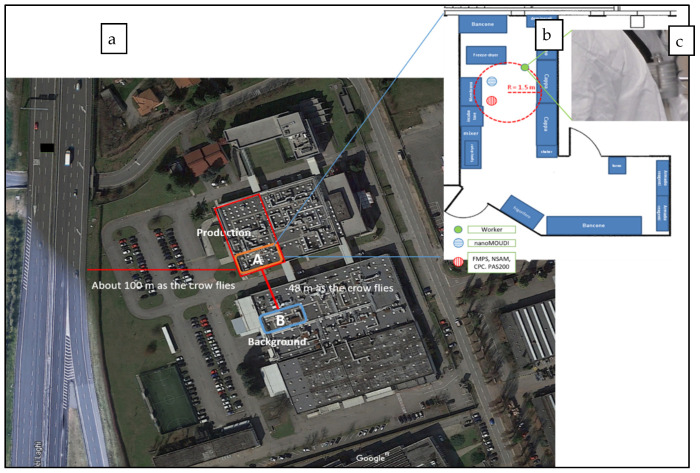
(**a**) Factory satellite view (Google Earth source), point A indicates the LTA NZ production laboratory and point B the room used for background monitoring; (**b**) LTA NZ laboratory floor plan with instruments location; (**c**) Sioutas impactor within the worker personal breathing zone.

The measurement strategy included: a walkthrough in the production site in order to obtain information about workplaces, collecting raw data of PNC during the production phases that would be useful to plan the experimental setup, and acquiring a small quantity of the produced LTA NZs for a powder trial sample. During the walkthrough, information about the overall exposure scenario was collected, not only related to the specificity of production phases (physical and chemical properties of materials and phases) but also to the main characteristics of the LTA NZ production laboratory, e.g., location, area, volume, use of collective and personal protective equipment (CPE and PPE), natural or forced ventilation, and working procedures. We collected background (Bkg) measurements in two rooms—A and B—on the ground floor of the factory, located in an industrial area far from about 100 m to a congested highway (Figure 1a). Room B was in a different building near the LTA NZ laboratory (room A). In room B, no working activities were performed during all LTA NZ production phases and moreover no products or materials traced back to the potential airborne particulate or secondary inorganic aerosol (SIA) [24] and SOA formation were present; forced ventilation was turned off and two different doors communicated with an internal hallway on one side, and with the outdoor in the other side. Room A was located in the LTA NZ laboratory in which production phases 0–4 (Table 1) were carried out; other chemical agents not strictly connected to LTA NZ production were also stored in special containers; only natural ventilation was present and it could largely contribute to the total PNC. A fume hood was present as CPE. The workers wore the PPE required by the internal safety procedures which included fire retardant antistatic and antiacid coats, protective gloves, protective goggles, and protective masks. For raw data collection, a handheld CPC was used to measure PNC in order to highlight the different concentration level between Bkg in room B, Bkg in room A, and some critical production phases performed with LTA NZ in powder form.

Simulation of the handling processes (racking, handling, pouring, and accidental spillage) of LTA NZ powder trial samples, were performed in a glove box (Figure S1) isolated from outside influences in order to significantly minimize the Bkg values and the possible external sources contributions [46]. Then, LTA NZ emission inside the glove box was collected by a Sioutas cascade impactor and analyzed by SEM for morphological characterization.

Finally, the extensive measurement campaign lasted two weeks of May. It was planned on the same season of the previous walkthrough in order to obtain comparable climatic conditions. During the first week, Bkg measurements were carried out in room B (Figure 1a) and in the second week measurements related the LTA NZ production phases were performed in room A (Figure 1a). All measurement slots lasted 8 h per day and both real-time and integration-time instruments were used. In order to compare the particle size distribution between room A and room B, the data recorded on a typical day of production in the room A, compared to those recorded on the same day of the previous week in room B, were analyzed. During the LTA NZ measurements in room A, the instruments were placed within a radius of 1.5 m from the operator (Figure 1b); a Sioutas impactor was worn by the worker during the production phases, placed within their personal breathing zone (PBZ) (Figure 1c).

In this study, we adopted two different approaches to distinguish LTA NZs from the background airborne particulate matter. The first one is a spatial approach known as background far-field (Bkg FF), while the second one is a time series approach called background near-field (Bkg NF), based on the principles deeply described by Kuhlbusch et al., 2011 [46] and Brouwer et al., 2012 [47].

The Bkg FF was measured in the room B, placed far from the laboratory where LTA NZs were produced (room A) but with the same structural characteristics. Otherwise, the Bkg NF was measured inside room A, before starting the production. We combined both the above-mentioned approaches in order to overcome limitations related to the availability of instruments which did not allow simultaneous measurements in room A and room B.

According to Asbach et al., 2012 [48] and Fonseca et al., 2016 [49], we assumed a PNC significant level (calculated as the mean Bkg NF PNC value plus three time the standard deviation) at the value above which the PNC level can be attributed to emissions during the production phases inside the LTA NZ laboratory.

The results obtained during the measurement campaign and the subsequent off-line analysis will be discussed below.

## 3. Results and Discussion

### 3.1. Real-Time Measurements

In Table 4, mean values and standard deviations of PNC, D_avg_, LDSA, and p-PAH are reported for Bkg FF and Bkg NF.

Since the PNC values of Bkg FF were always lower than PNC of Bkg NF, we referred to the second one to calculate PNC significant level (5152 #/cm^3^) in the worst exposure scenario, according to a precautionary approach.

**Figure 2:**
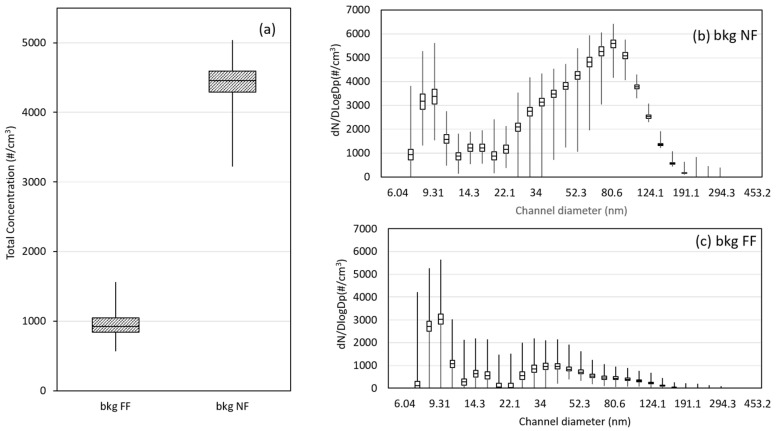
Box plots of (**a**) CPC total number particle concentration of near field (Bkg NF) and far field background (Bkg FF); Mean PSD for (**b**) Bkg NF and (**c**) Bkg FF.

Both particle size distributions of Bkg FF and Bkg NF show essentially a bi-modal distribution. For the Bkg FF, the first mode occurs in the particle size range with diameter centered at about 10 nm (nucleation mode, NM less than 30 nm) and the second one in the range of particle diameter greater than 30 nm (accumulation mode, AM) [50]. Although the first feature is about the same for Bkg NF and Bkg FF; the second one has the modal value at about 36 nm for the Bkg FF and at about 100 nm for the Bkg NF (Figure 2b,c) showing a shift towards the dimensions close to the produced NZs’ typical size. Such figures confirm the contribution to the Bkg NF related to the production activities as already highlighted in previous preliminary analysis [51].

**Figure 3:**
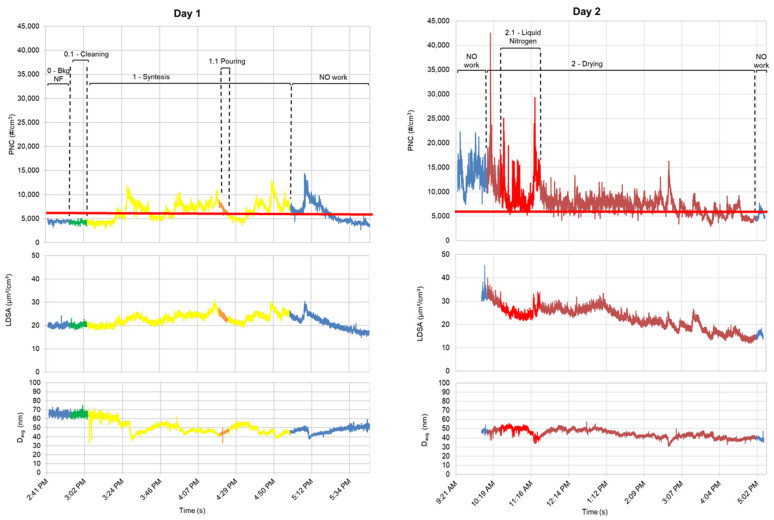

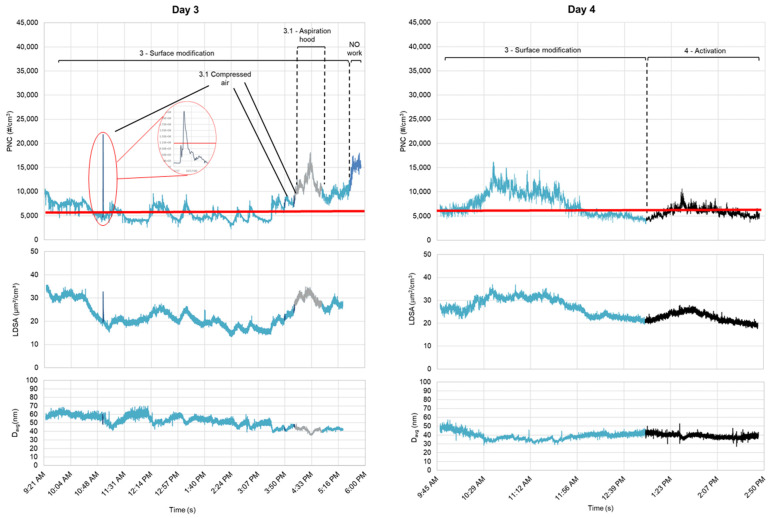
PNC, LDSA, and D_avg_ time series during LTA NZ production phases. The red line indicates the PNC significant value.

Real-time measurements of LTA NZ production phases of cleaning, synthesis, pouring, drying, and surface modification were monitored with respect to the significance level (red line in Figure 3) of 5152 #/cm^3^.

During synthesis and pouring phases (day 1) and surface modification and activation phases (day 4), PNC values frequently exceeded the significance level and a decrease in D_avg_, obtained by FMPS measurements was correspondingly observed (Figure 3). The decrease in average diameter of airborne particles corresponding to a simultaneous increase in PNC refers to an increase in contribution of smaller particles in the same amount of volume during this process phase. As a consequence, an emission of particles with an average size of less than 60 nm can be supposed.

During the drying phase (day 2) and surface modification phase (day 3), PNC values also exceeded the significance level when liquid nitrogen was used (drying phase) and when compressed air was used during cleaning activities to remove the LTA NZs from to the equipment (surface modification phase). High values of PNC can be attributed not only to the LTA NZ deposited on the equipment, but also to an effect of raising dust from the surrounding surfaces. It should be stressed that, in general, PNC could also be influenced by the air relative humidity (RH) [9], showing a well-recognized trend compared to the high frequency variability of PNC during working activities (e.g., compressed air use). In particular, on day 3 the contribution of RH to PNC is not relevant because the RH time course has a monotone decreasing trend (see Figure S5): the maximum value of PNC during the compressed air use at 4:21 PM corresponds to the daily minimum RH values.

The insert in Figure 3 shows the increase in PNC (max. value about 20,000 #/cm^3^) in the surface modification phase (day 3) when compressed air was used out of the fume hood. Again, it was observed that an increase in PNC corresponds to a decrease in the average particle size from 60 nm to about 40 nm.

PNC values at the beginning of each production phase are 4423 (±243) #/cm^3^, 12,771 (±2421) #/cm^3^, 8588 (±949) #/cm^3^, and 6142 (±472) #/cm^3^, respectively. The higher PNC values referring to the second, third, and fourth day can be due to particles infiltration from outdoor (as confirmed by p-PAH and RH% levels in Table 4; Table 5 respectively) and to emissions during the production phases related to the previous days, since PNC values return to the levels of the first day after the fume hood was turned on. Moreover, LDSA and PNC time series have the same trend. In particular, LDSA values in room A are four times higher than those recorded in room B (Table 5): these values also are in agreement with the 8h-TWA calculated by the instrument. It is worth noting that LDSA values were acquired not simultaneously in room A and B; in any case, the same time period (four days including 68,125 valid counting) was used. In addition, room A and room B are at the same distance from the highway crossing near the factory and they are equally permeable to external emission sources.

Therefore, high values of LDSA in room A can be likely associated to the production, based on the fact that during the four monitoring days anthropogenic activities near the facility—such as gardening activities, works of loading and unloading goods, building maintenance activity, or other ordinary and extraordinary activities—have not occurred.

Since microclimatic parameters may also influence the measurement results, in Table 5 the mean value of RH and ambient temperature on the four production days in room A and B are reported. RH is twice higher in room A than in room B, probably due to worsening of weather conditions (rainy days) occurring during the measurement in room A.

**Figure 4:**
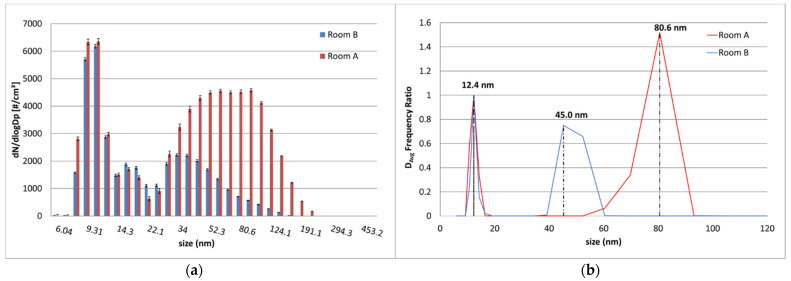
(**a**) Comparison of the median particle size distributions and (**b**) D_avg_ frequency normalized referred to room A (red tones) and room B (blue tones).

Moreover, particle size distributions of the median values on the third day of monitoring for both rooms A and B have been compared in Figure 4a. As a hypothesis, both distributions can be assumed as bi-modal: the first feature (NM) can be likely related to environmental background [50] for both rooms A and B; the second one (AM) highlights the different contribution probably related to the working activity in room A [52,53].

D_avg_ frequency values (Table S2) also confirm the above considerations: D_avg_ frequency values for NM is about 12.4 nm in room A and in room B; D_avg_ frequency values for AM in room A is 80.6 nm and in room B is 45.3 nm. In Figure 4b, D_avg_ frequency values normalized respect to NM are reported. In room A, the mean particle size of AM is in according with the typical size of produced LTA NZs, as confirmed by the dynamic light scattering (DLS) analysis provided by the manufacturer (Figure S2).

**Figure 5:**
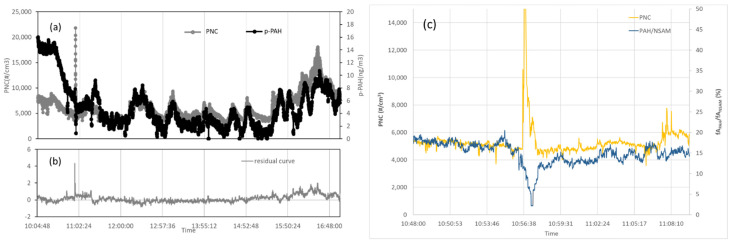
(**a**) Time course of PNC (grey curve) and p-PAHs concentration (black curve) of day 3; (**b**) Residual curve after interpolation and background subtraction; (**c**) Diffusion charging measurements (ratio fA_PAH_/fA_NSAM_) and PNC signal.

In order to evaluate the influences by outdoor pollutants, on day 3 the PNC values during the cleaning activities with compressed air and the p-PAH outdoor contribution as interfering agent to PNC measurements were compared. A critical evaluation has been carried out by comparing data from four different devices: CPC, PAS2000, NSAM, and FMPS (Figure 5).

Before 11:02 a.m., a high intensity peak can be seen in the PNC curve in correspondence to the cleaning operation using compressed air (very narrow for about 60 s).

To deeply investigate the nature of this spillage, we used the PAS2000 and NSAM devices based on the diffusion charge. In both instruments, the particles are superficially charged: NSAM generates a corona discharge on the particles in the sampling flow whereas PAS2000 generates the charge by UV radiation and it is selective for particles on which are adsorbed p-PAH or, to a lesser extent, carbon based particles (i.e., soot). The ratio between the two current signals (fA_PAH_/fA_NSAM_) is high if the airborne particulates contain p-PAH, mainly due to vehicular traffic and human activities; otherwise, the same ratio is low in the case of particulates containing non-UV reactive substances, such as salts, metals, or powders in the AM coated by nitrate, sulfate, water, hydrocarbons, or volatile species [54].

In Figure 5c, the ratio fA_PAH_/fA_NSAM_ in correspondence with the activity using compressed air is about 2%, showing that the eventual spillage is composed of particles that are non-UV reactive. Otherwise, before and after the spillage, the nature of the particulate is UV reactive and probably traced back to outdoor environment (p-PAH or carbon-based materials). More clearly, the considered event is so fast since it excludes emission sources not due to the work activity.

### 3.2. Off-Line Analysis

Teflon filters (22 pieces) collected during the production phases, were analyzed by means of ICP-MS technique referring to USGS method [55]. Samples in room A and room B were collected on 11 collection stages for size particles ranging from 0.056 µm to 18 µm. Elemental analysis was performed by inductively coupled plasma mass-spectrometry (ICP-MS) detection: the isotopes Be, B, Mg, Ti, V, Cr, Fe, Ni, Cu, Zn, As, Mo, Cd, Sb, Ba, Pb, and Si were measured. The microwave-assisted digestion method, preparation of working standard solutions and set up of analysis plasma with reaction cell parameter (Table S1), have been reported in Supporting Materials.

**Figure 6:**
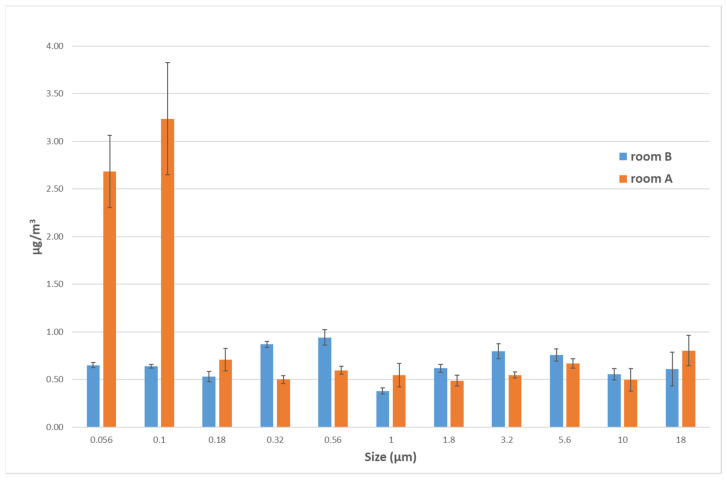
Comparison between room B (blue) and room A (orange) of airborne Si mass concentration in NanoMOUDI stages for size range from 18 µm to 56 nm analyzed with ICP-MS.

The Figure 6 shows Si ions mass concentration (µg/m^3^) respect to the powder sampled on each one of the 22 collection stages, in room A and in room B. In the stages that collect particles in the range of 0.18 to 18 µm, the Si ions concentrations are comparable in room A and room B.

A characteristic Si contribution related to airborne particles with dimensions in the range of 0.056 to 0.1µm is evident in the LTA NZ production laboratory (room A); this contribution is about four-times greater than the value obtained in room B, as in the case of LDSA previously described. It is worth noting that the Si ions concentrations can be indicative of spilled LTA NZs during the production phases. The histogram in Figure 6 highlights that the sum of mass concentrations related to the aerodynamic diameters of 0.056 and 0.1 µm subtracted by the background, is 4.63 µg/m^3^. This value may be intended as the upper limit of the mass concentration of Si-based airborne NMs with aerodynamic diameter <100 nm to which the worker is potentially exposed during one week of production process. In any case, this value is lower than the available occupational exposure limit for bulk amorphous silica recommended by the U.S. NIOSH (6 mg/m^3^ TWA per day) [19], and also lower than the occupational exposure limit for SiO_2_ NMs proposed by WHO (0.3 mg/m^3^ 8 h OEL for the respirable fraction) [56].

All of the other metal ion concentrations analyzed (see Figure S4) are comparable in room A and B, except for iron (Fe) ion concentration; this fact could probably be due to other work activities not strictly related to the LTA NZ production phases.

**Figure 7:**
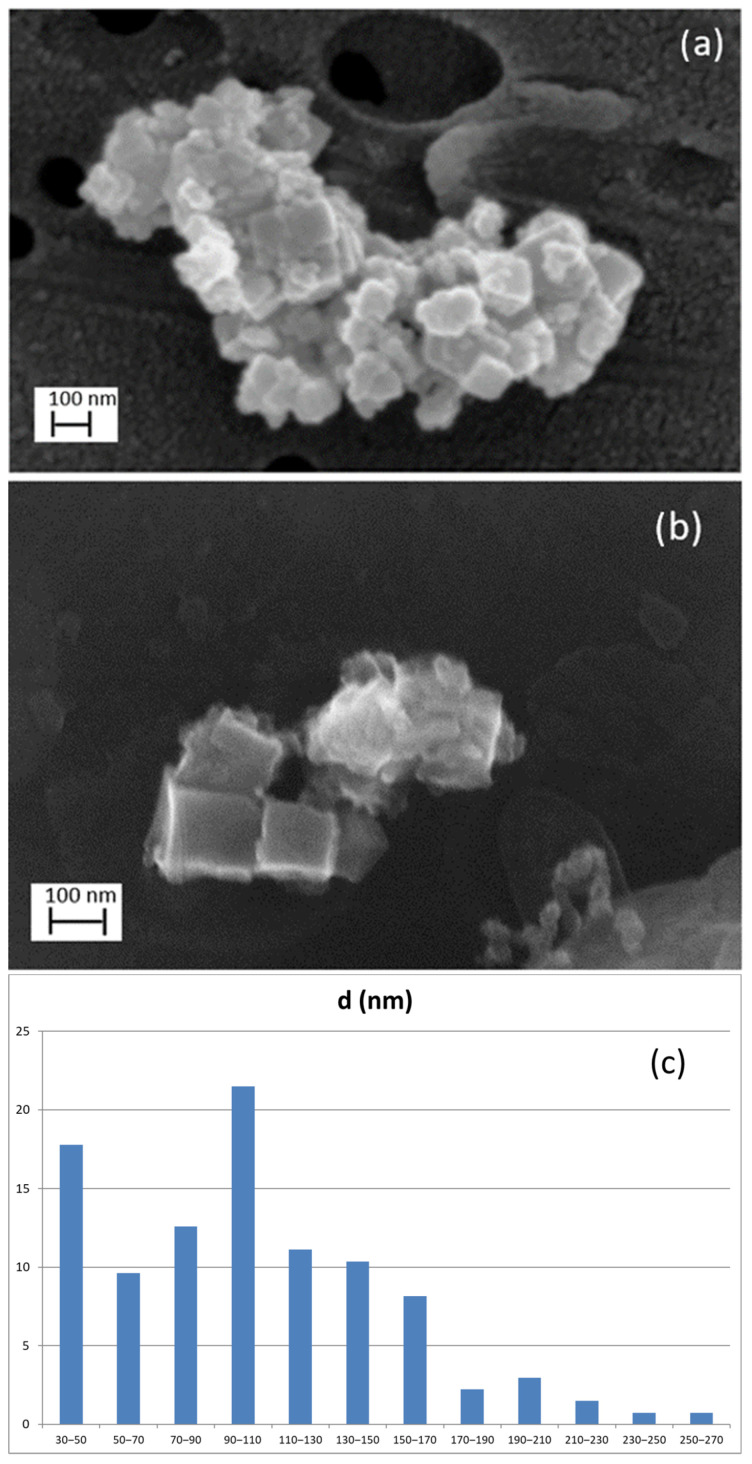
SEM image of LTA NZs: (**a**) trial sample as collected on aluminum filters during the laboratory simulations and (**b**) collected by Sioutas in the workplace. (**c**) Histogram of 150 particles diameter as measured in the trial sample.

The dimensional distribution of the LTA NZ trial sample collected on aluminum filters by Sioutas cascade impactor, during the laboratory simulations inside the glove box, was studied by examining the average diameter of about 150 particles using FEG SEM: the characteristic cubic shape morphology was easily recognized (Figure 7a). These particles are those measurable as distinguishable as single or within the aggregates by choosing about 50 random SEM fields within the filter: the mode was found to occur at 96 nm, the mean value at 105 nm, the value of the minimum diameter at 32 nm, and the value of the maximum diameter at 295 nm (Figure 7c). These values are in agreement with those reported by the manufacturer in Table 1.

SEM image of airborne materials collected in the workplace on the aluminum filter stage in the range of 56 nm to 100 nm (Figure 7b), also highlights the characteristic nanoparticles’ cubic shape with a lateral size of about 100 nm.

**Figure 8:**
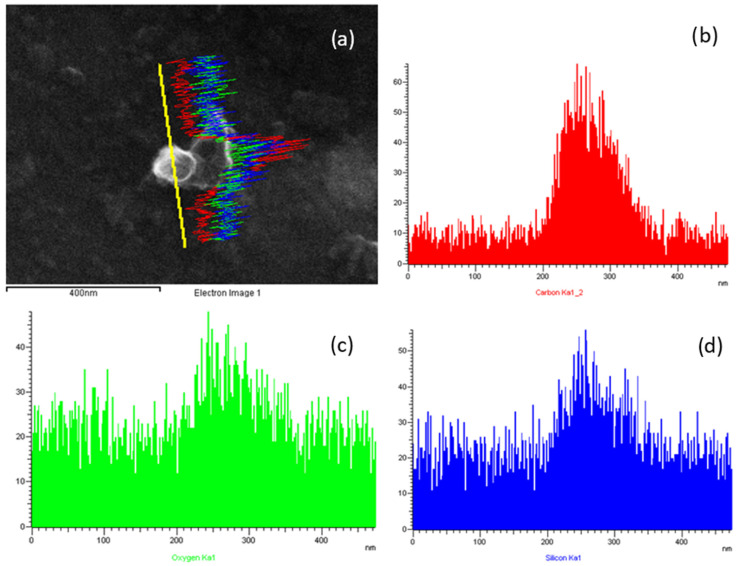
SEM image of LTA NZs particle (**a**) with the EDX signals (line scan mode) of carbon (red curve) (**b**), oxygen (green curve) (**c**) and silicon (blue curve) (**d**), collected by nanoMOUDI in the workplace.

Moreover, EDX analysis on a single cubic shaped particle (Figure 8) remarkably reveals Si and oxygen (O) signals that are the typical chemical elements of LTA NZs; more deeply, in line-scan mode the increasing of signals related to O and Si can be observed just close to the particles. Furthermore, carbon (C) signal increases are probably due to volatile compounds coated on particles (Figure 8b).

In conclusion, the characteristic cubic shape and the chemical characterization reveal that airborne LTA NZs were collected during the production phases.

### 3.3. External Source Interactions with the Background

The release mechanisms of airborne NMs may be influenced not only by the process, by the NM’s nature and by the different emission sources in the workplace, but also by the environmental conditions [57,58] in terms of climatic parameters and environmental pollutants, including the possible formation of SOA and SIA ultrafine structures [59,60].

In order to highlight the influence of environmental outdoor ultrafine particles (UFPs) to the PNC measured inside the LTA NZ laboratory (room A), our study focuses on day 5 when the external gardening activity happened.

**Figure 9:**
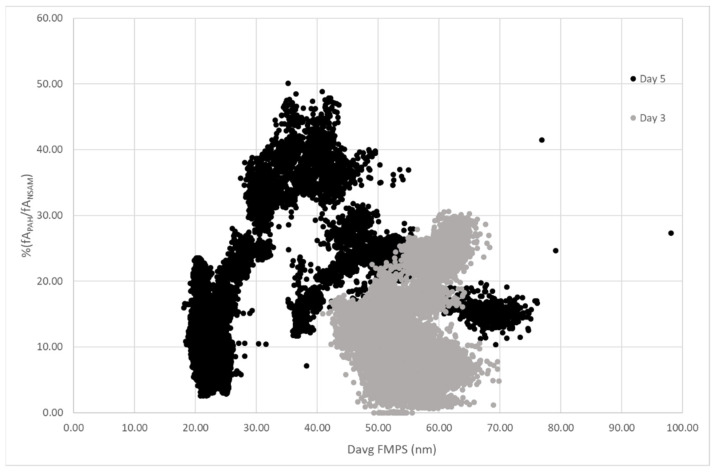
% fA_PAH_/fA_NSAM_ vs. D_avg_ (FMPS) plots for sampling during day 3 (surface modification) and day 5 (gardening activity).

In Figure 9, the fA_PAH_/fA_NSAM_ ratio versus the average diameter obtained by FMPS is reported for day 3 and day 5. Day 5 shows the characteristic triangular shape where the three apices are related to nucleation mode particle contribution and to accumulation mode due to ‘fresh’ and ‘old’ diesel aerosol particles being emitted. According to Bukowieki et al., (2002) [54], fresh AM particles are shown in the apex of the triangle and the lack of points in the triangular central part indicates the proximity to the emissive source (day 5). The same analyses were carried out on the all LTA NZ production days and we reported in Figure 9 the day 3 as an example (days 1, 2, 4 are reported in Figure S3). The central area of the triangle referring to day 3 is concentrated between D_avg_ from about 40 nm to 70 nm and the triangle apexes are not well defined. This distribution highlights a low value of the percentage fA_PAH_/fA_NSAM_ ratio, mainly due to the contribution of particles coming from vehicular traffic which—being enough far from the experimental sampling point—can be able to adsorb or condensate non-photoemissive materials.

It is worth noting that D_avg_ used in our study is calculated by FMPS, not derived from NSAM [54], thus resulting in fA_PAH_, fA_NSAM_, and D_avg_ as three independent variables.

## 4. Conclusions

An integrated multi-parametric approach was used to compare environmental and occupational parameters (PNC, D_avg_, Size distribution, LDSA, p-PAH, RH%, and ambient temperature) during the production phases of LTA NZs in a production site. Real-time measurements have been integrated with time-integrated personal and environmental samplings; off-line techniques were subsequently used to characterize the collected airborne nanomaterials by ICP-MS and SEM-EDX.

The main emission sources of nanoparticles within the working environment have been identified. Events due to LTA NZ spillage in the air during the work procedures have been characterized and isolated from those due to the external sources. In fact, on-line measurements campaign showed a high contamination due to external emission sources—such as vehicular traffic and anthropogenic activities—that took place near the production site. The combined use of data collected with PAS2000, NSAM, and FMPS could be used to identify both the composition and the origin of the main contaminants, and to distinguish the fresh emissions from the old ones and, consequently, the distance from the emission sources.

During the production, in the cleaning phases, PNC exceeded the significant value twice when compressed air was used. Subsequently, SEM-EDX and ICP-MS analyses on the airborne particles collected during the production phases could be used to identify the nature and the morphology of the leakage, associated with the produced LTA NZs. Moreover, it is worth noting that—during the LTA NZ production—the value of occupational parameter LDSA, corresponding to the 8h-TWA, is about four times higher than the background one (Table 5); this ratio is confirmed by the Si ions concentration determined by ICP-MS in the dimensional range of the produced LTA NZs.

These last two results represent evidence of the release of airborne LTA NZs in the working environment; however, the mass concentration of Si-based particles collected during the production is lower than the reference values now recommended for Si-based NMs exposure in the workplace.

Although the cleaning activities by using compressed air have been clearly identified as emission sources due to the raising dust from the surfaces, the exceeding of significance level values in all monitored stages of production cannot exclude LTA NZ exposure during other working procedures.

Furthermore, since toxicological studies on LTA NZ trial sample materials reported mild—but detectable—early genotoxic and inflammatory effects on human alveolar epithelial cells, recommendations for workers’ health protection should be allowed. In particular, a specific re-design of workstations to isolate workers from the production environment, also using automated enclosed systems, may be recommended. In any case, an effective forced ventilation system integrated with the CPE and PPE use, as is already done, will contribute to risk mitigation.

In conclusion, it will be important to carry out toxicological studies also on filters sampled during the working phases in order to better investigate the possible effects on human health of airborne NMs present in the workers’ PBZ, which certainly includes the produced LTA NZs.

## Figures and Tables

**Table 1:** LTA NZs monitored production phases.

Phases		Processing Time	No. of Workers	Physical State
0	Cleaning	10 min		
0.1	Generic cleaning phase (compressed air involved)		2	Powder form (in processing residues)
1	Synthesis	24 h		
1.1	Preparation (surface and equipment cleaning)	10 min	1	Powder form (in processing residues)
1.2	Bulk matter dispersed in aqueous solution		1	Liquid suspension
1.3	Liquid suspension distribution in closed bins	1 h 30 min	1	Liquid suspension
2	Drying	48 h		
2.1	Preparation (surface and equipment cleaning)	10 min	2	Powder form (in processing residues)
2.2	Closed system employed: spray drying or freeze drying	2 h	2	Liquid suspension
2.3	Sublimation	1 h	2	Powder form
3	Surface Modification	24 h		
3.1	Preparation (surface and equipment cleaning), transfer of incoming materials	1 h	1	Powder form (in processing residues) and liquid suspension
3.2	Start phase (bulk matter suspension in organic solvent)	30 min	1	Liquid suspension
3.3	Finish phase (transfer of incoming materials and equipment cleaning)	30 min	1	Powder form
3.4	Laboratory oven drying in specific bins	12 h *	1	Powder form
4	Activation	24 h		
4.1	200 °C vacuum treatment		2	Powder form

* Exposure time 30 min.

**Table 2:** Set-up and characteristics of the instrumentation used for measurements and samplings.

Instrument	Class	Principle of Operation	Output	Size Range (nm)	Time Resolution (s)	Flow (L/min)	Detection Limits	Accuracy
CPC TSI Inc. Mod. 3007	Real time	Optical detection	PNC (#/cm^3^)	10–1000	1	0.7	1–1 × 10^5^ #/cm^3^	±20%
FMPS TSI Inc. Mod. 3091	Real time	Electrical mobility	PNC (#/cm^3^) Size distribution	5.6–560	1	10	Small part.: 100–1 × 10^7^ #/cm^3^ Large part.: 1–1 × 10^5^ #/cm^3^	±15% MDC *
NSAM TSI Inc. Mod. 3550	Real time	Diffusion charging	Surface area running avg (µm²/cm³) and total (µm²) TB or A fractions	10–1000	1	2.5	TB: 0–2500 μm^2^/cm^3^ A: 0–10,000 μm^2^/cm^3^	±20%
PAS2000 EcoChem Inc.	Real time	Photoelectric Ionization	p-PAH (ng/m^3^)	10–1000	10	2	>3 ng/m^3^	±30%
nanoMOUDI MSP Mod. 122 R	Time-integrated area sampler	Aerodynamic diameter	Particle gravimetric mass Size distribution Samples for off-line analysis	10–18,000	-	30	-	-
SIOUTAS SKC Ltd.	Time-integrated personal sampler	Aerodynamic diameter	Particle gravimetric mass Size distribution Samples for off-line analysis	250–2500	-	9	-	-

* Mean Diameter Counting (MCD) vs. SMPS TSI/3936 for particles <100 nm of polystyrene latex (PSL), according to Asbach et al., 2009 [45].

**Table 3:** LTA NZ physicochemical characteristics declared by the manufacturer.

Technical name	Submicrometer LTA Zeolite
Physical state	Powder, dispersion in organic matrix
Chemical composition and surface coating	Na_8_ Al_8_ Si_8_ O_32_ with surface aromatic silanes coating based
Crystalline structure	Orderly crystalline structure
Physical shape/aspect	Cubic shape
Dimensions	60% n/n < 100 nm determined by dynamic light scattering (DLS) analysis
Surface area	819.6 m^2^/g determined by Brunauer–Emmett–Teller (BET) analysis
Density	1.8 g/cm^3^
Porosity	0.4352 cm^3^/g Total pore volume determined by BET analysis

**Table 4:** Mean and standard deviation of PNC, D_avg_, LDSA, p-PAH in Bkg FF and Bkg NF.

	Bkg FF 13:00–17:00		Bkg NF 14:42–15:00	
	Mean Value	Std.Dev.	Mean Value	Std.Dev.
PNC (#/cm^3^)	956	155	4423	243
D_avg_ (nm)	36	3	65	2
LDSA (µm^2^/cm^3^)	3.3	0.6	19.9	0.7
p-PAH (ng/m^3^)	1.5	0.7	2.3	0.7

**Table 5:** LDSA and microclimatic parameters for rooms A and B.

	Room A		Room B	
	Mean Value	Std.Dev.	Mean Value	Std.Dev.
LDSA (µm²/cm³)	23	5	6	3
T (°C)	23.5	0.5	20.7	0.6
RH (%)	44	7	24	4

## Data Availability

Not available.

## Supplementary Materials

The following supporting information can be downloaded at: https://www.mdpi.com/article/10.3390/nano12091448/s1, Figure S1: Glove-box experimental set up; Table S1: Set up of analysis plasma and reaction cell parameter; Table S2: D_avg_ frequency values of particle size distribution in day 3 for room B and room A; Figure S2: LTA NZs Dynamic Light Scattering analysis provided by the manufacturer; Figure S3: PAS/DC versus D_avg_ (FMPS) plots for sampling during days 1, 2 and 4; Figure S4: ICP-MS analysis on nanoMOUDI stages for 17 metals; Figure S5: PNC and Relative humidity time course during the Day 3 during surface modification phase.
