# Supplementary material for: Airborne LTA Nanozeolites Characterization during the Manufacturing Process and External Sources Interaction with the Workplace Background
Source: Nanomaterials (Basel). 2022 Apr 24;12(9):1448. doi: 10.3390/nano12091448 (PMC9104400; doi:10.3390/nano12091448)
Supplement: Supplementary file 1 [file nanomaterials-12-01448-s001.zip › nanomaterials-1600716-supplementary.pdf]

Supplementary Material

ICP analysis metod.

Each sampling filter was placed in PTFE vessels. 4 mL of 67–69% HNO3 (Ultrapure-for trace analysis, Carlo Erba Reagent), 2 mL of 47 – 51% HF (Ultrapure-for trace analysis, Carlo Erba Reagent) and 2 mL of 30–32% H2O2 (Ultrapure-for trace analysis, Carlo Erba Reagent) were added to each vessel; the vessels then were placed in a microwave (MW) oven (Milestone Ethos Touch Control with HPR 1000/10 rotor) and digested in a four-step temperature-time program. The temperature linearly increases for the first step with a power of 600 W up to 180 °C in 15 min; for second step the temperature linearly increases with a power of 600W up to 240°C in 5 minutes and then at 240°C for 15 minutes. The solutions were then analyzed by ICP-MS (Agilent Technologies 7500 ce). Standard solutions (Multistandard 26 elements, Ultra scientific, North Kingstown, United States), for daily calibration, were matrix-matched by preparation in HNO 32% (w/w).

To control nebulizer efficiency, an internal standard (cesium, 50 ng/ml) was used. The analysis plasma and reaction cell parameter are reported in table S1.

Table S1. Set up of analysis plasma and reaction cell parameter

**Plasma parameters**	
RF Pawer	1500 W
Carrier Gas	0.88 L/min
Makeup Gas	0.20 L/min
Nebulizer Pump	0.10 rps
S/C Temp	2 degC
Reaction Cell parameter	
H_2 Gas	4.0 mL/min

The glove box has a volume of 0.5 m^3 and it is equipped with ball valves. An HEPA filter is used for external insulation; a solenoid valve allows to preserve internal isobaric conditions, while a fan, with speed controller, can rotate over 360°. The fully equipped glove box is shown in supplementary material

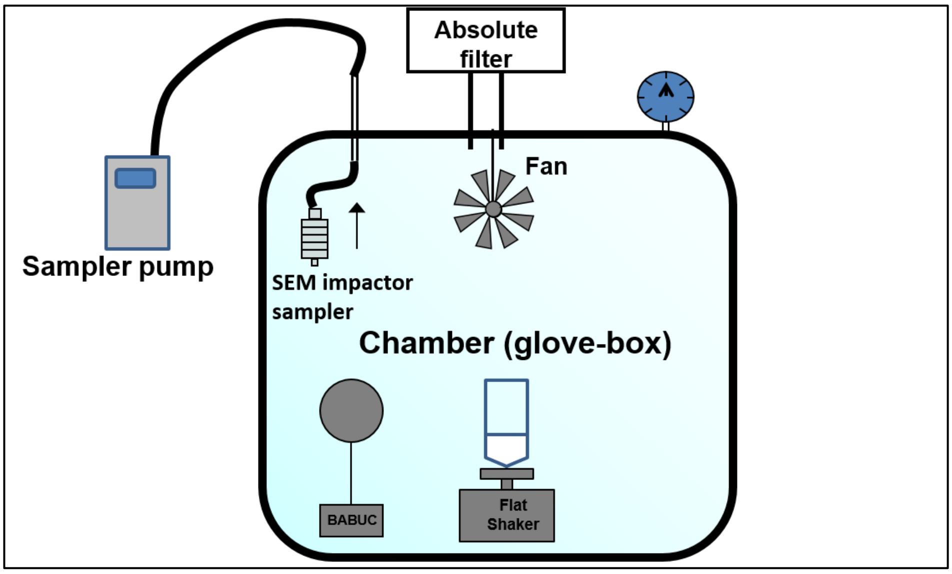

Figure S1. Glove-box experimental set up.

Table S2. Davg frequency values of particle size distribution in day 3 for room B and room A

Size nm	Davg room A Frequency	Davg room B Frequency
**6.04**	0	0
**6.98**	0	0
**8.06**	0	0
**9.31**	15	29
**10.8**	8840	4960
**12.4**	14958	20246
**14.3**	4969	3129
**16.5**	18	435
**19.1**	0	1
**22.1**	0	0
**25.5**	0	0
**29.4**	0	0
**34**	0	0
**39.2**	0	182
**45.3**	0	15182
**52.3**	0	13363
**60.4**	960	61
**69.8**	5071	5
**80.6**	22721	1
**93.1**	46	4
**107.5**	2	1
**124.1**	0	1
**143.3**	0	0
**165.5**	0	0
**191.1**	0	0
**220.7**	0	0
**Count**	57600	57600

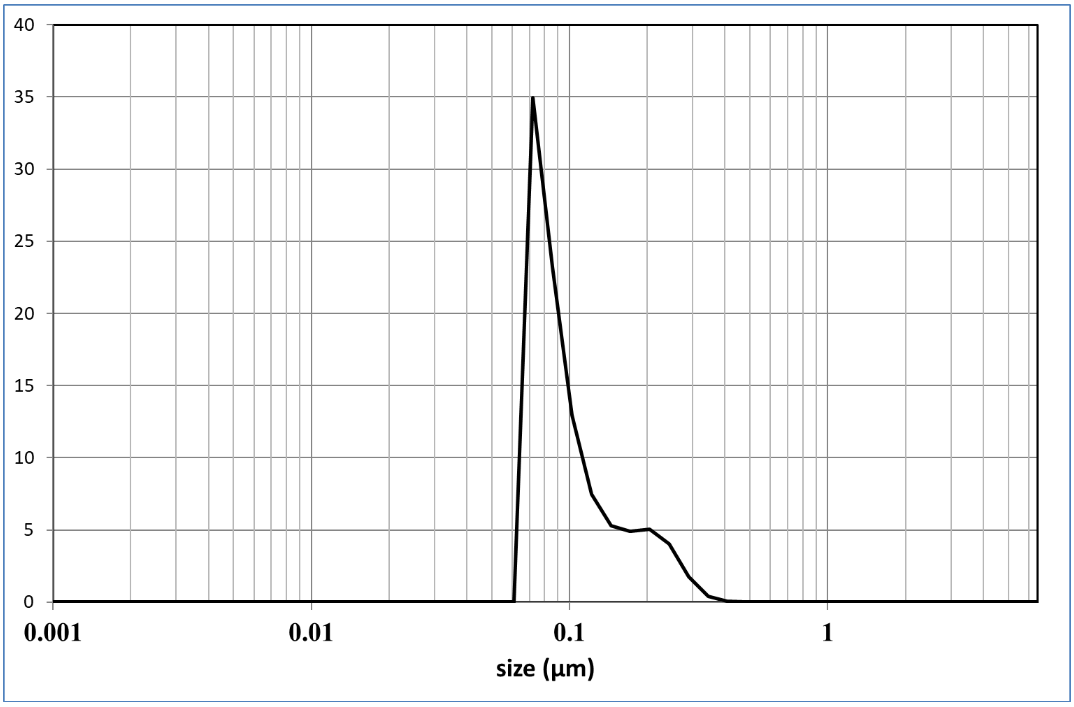

Figure S2. LTA NZs Dynamic Light Scattering analysis provided by the manufacturer.

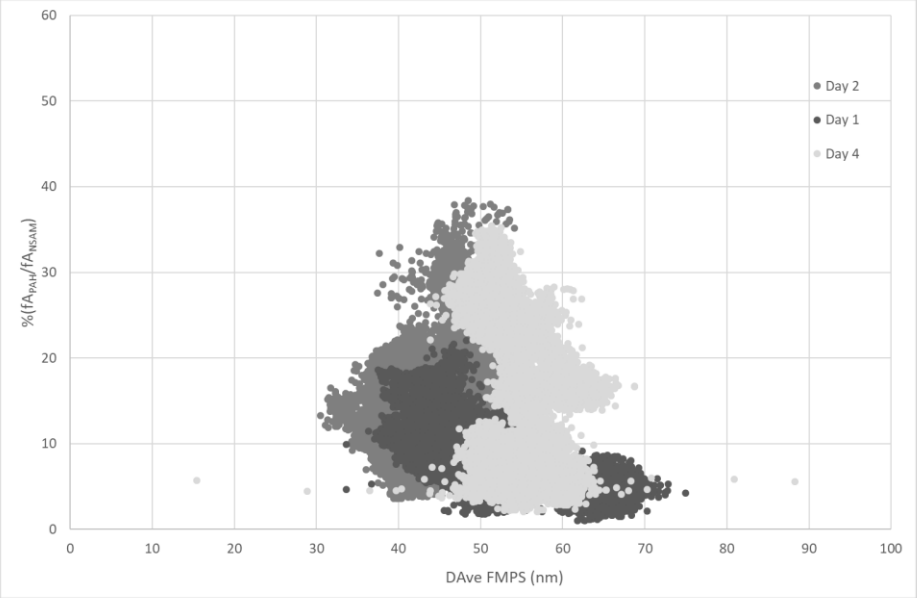

Figure S3. PAS/DC versus D_{avg} (FMPS) plots for sampling during days 1, 2 and 4

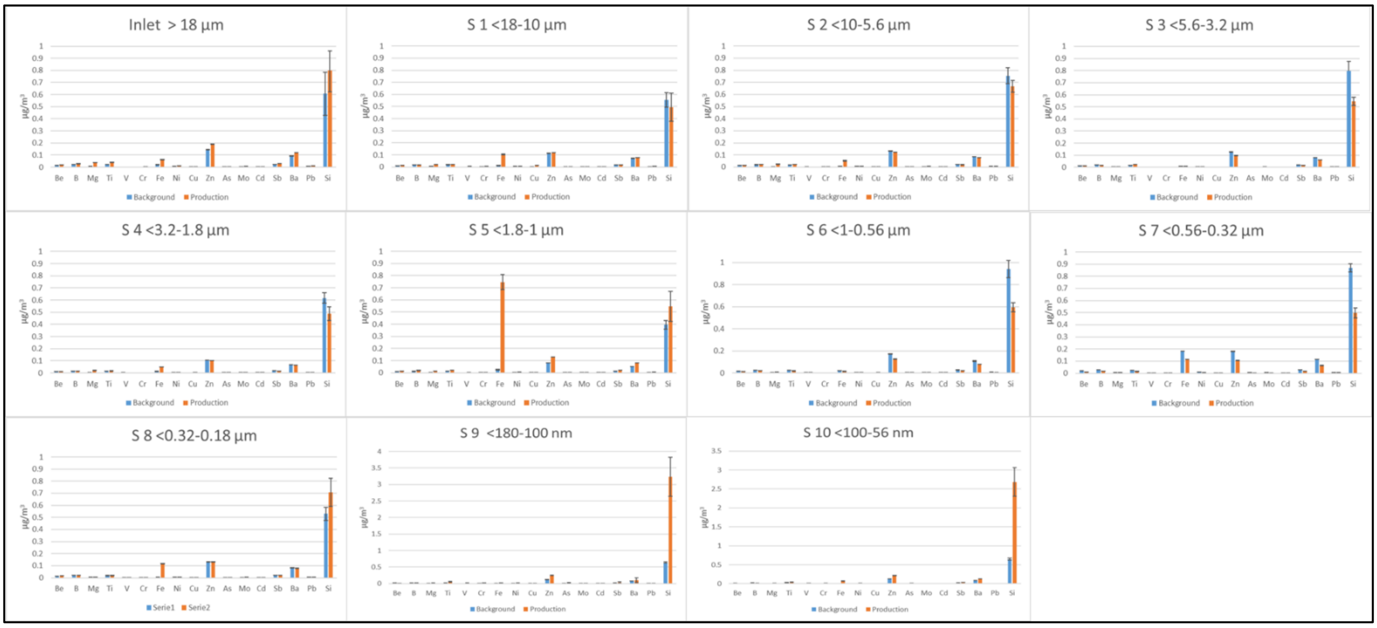

Figure S4. ICP-MS analysis on nanoMOUDI stages for 17 metals

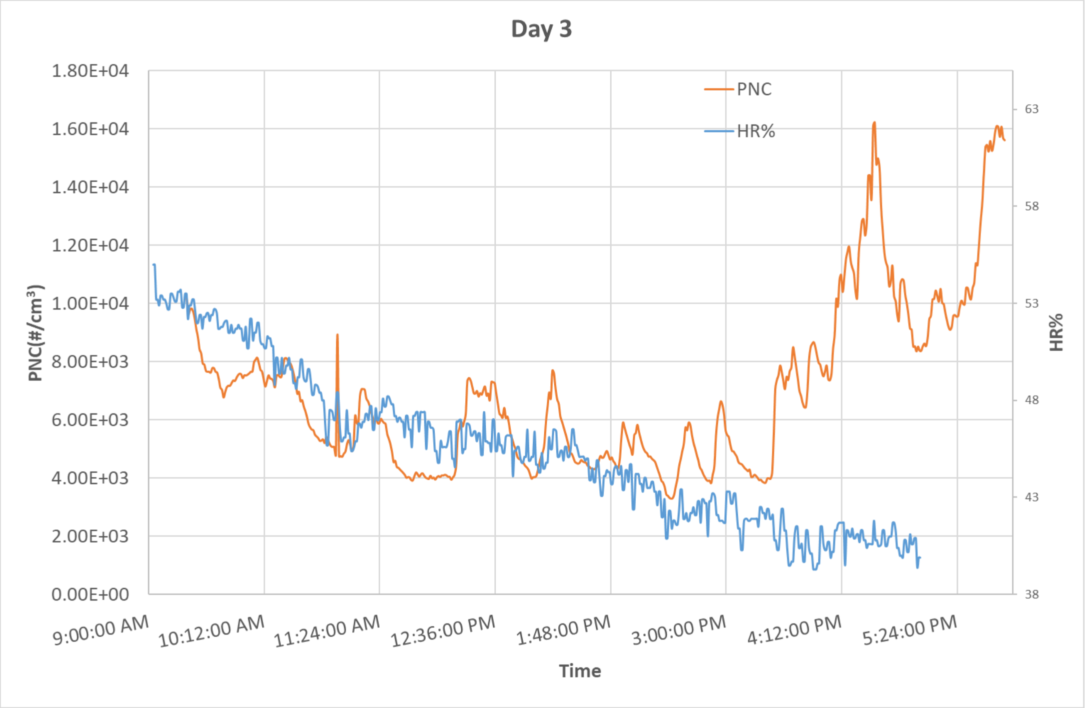

Figure S5. PNC and Relative humidity time course during the Day 3 during surface modification phase
